# Tumor invasion to the spinal region after primary surgery: A case report

**DOI:** 10.22088/cjim.12.0.467

**Published:** 2021

**Authors:** Zahra Davoudi, Arezoo Chouhdari, Guive Sharifi, Nader Akbari Dilmaghani

**Affiliations:** 1 Skull Base Research Center, Loghman Hakim Medical Center, Shahid Beheshti University of Medical Sciences, Tehran, Iran; 2 Social Determinants of Health Research Center, Amir-Al-Momenin Hospital, Tehran Medical Sciences Branch, Islamic Azad University, Tehran, Iran; 3 Department of Neurosurgery, Loghman Hakim Hospital, Shahid Beheshti, University of Medical Sciences, Tehran, Iran; 4 Hearing Disorders Research Center, Shahid Beheshti University of Medical Sciences, Tehran, Iran; 5 Department of Otolaryngology, Head and Neck Surgery, Loghman Hakim Hospital, Shahid Beheshti University of Medical Sciences, Tehran, Iran

**Keywords:** Pituitary carcinoma, Tumor invasion, Cushing syndrome

## Abstract

**Background:**

It is believed that pituitary carcinoma is a rare disorder and arise from the transformation of benign invasive macroadenomas, and the process of this transformation takes place slowly.

**Case Presentation:**

A 51-year-old man presented with the clinical features of Cushing syndrome and walking impairment who was diagnosed with metastatic corticotroph pituitary carcinoma to the spine region, 6 years after the initial resection of a primary invasive pituitary adenoma. He made a visit to neurosurgery and endocrinology clinic with the chief complaint of weight gain, facial and extremities swelling, paresthesia, weakness, motion and speaking impairments, and HTN which all appeared through the last 1 year; hormonal laboratory tests showed urine free cortisol (UFC) 197.8 and 367. 30 ug/24hrs (36-137), cortisol 8 am after 1 mg overnight dexamethasone test 375 ng/mL (50-250) and ACTH 59 pg/mL. MRI study revealed a mass in the brainstem with the compression effect on spinal region, pituitary imagine does not differ from the last MRI. He underwent a neurosurgery for spinal mass resection, which was successful and the total mass was resected. After surgery, the patient's condition became better.

**Conclusion:**

Pituitary carcinoma is a rare entity impossible to recognize as a primary tumor because its diagnosis by definition requires the presence of metastasis. Clinical awareness of the rare possibility for aggressive adenomas will progress, to metastasize is essential to appropriately monitor patients for possible early detection and treatment of pituitary carcinoma.

Pituitary adenomas constitute 10% to 15% or intracranial neoplasms. Most of the pituitary tumors are noninvasive, but up to 20-25% of pituitary adenomas are invasive and invade the cavernous sinus, sphenoid sinus and bone (1, 2). Pituitary carcinoma is a rare disorder accounting for 0.1-0.2% or pituitary tumors. It is believed that pituitary carcinomas arise from the transformation of benign invasive macroadenomas and the process of this transformation takes place slowly (3). A large majority of pituitary carcinomas preserves a capacity for producing anterior pituitary hormones, most notably ACTH and prolactin (4). However, a definite distinction between pituitary carcinoma and adenoma is impossible on the basis of endocrinological, neuroradiological, and histological criteria. The presence of CNS and/or distant metastases is a prerequisite for establishing the diagnosis of this clinical entity (5). Nevertheless diagnosis of invasive-secretory pituitary carcinoma is rare, and usually can be mistaken with other non-related conditions which may lead to malpractice and maltreatment. We reported a case of corticotroph carcinoma which presented initially as an invasive macroadenoma.

## Case presentation

**Figure 1(A, B):**
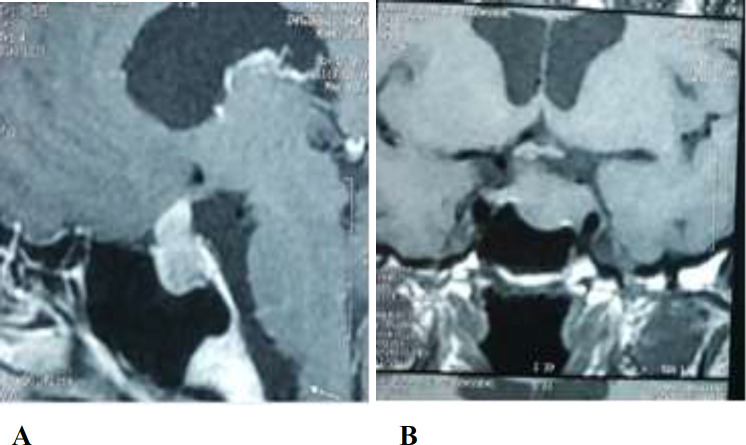
Preoperative T1 imaging (MRI) of the sellar mass (sagittal A, coronal B, view) demonstrating pituitary macro adenoma

**Figure 2 (C, D):**
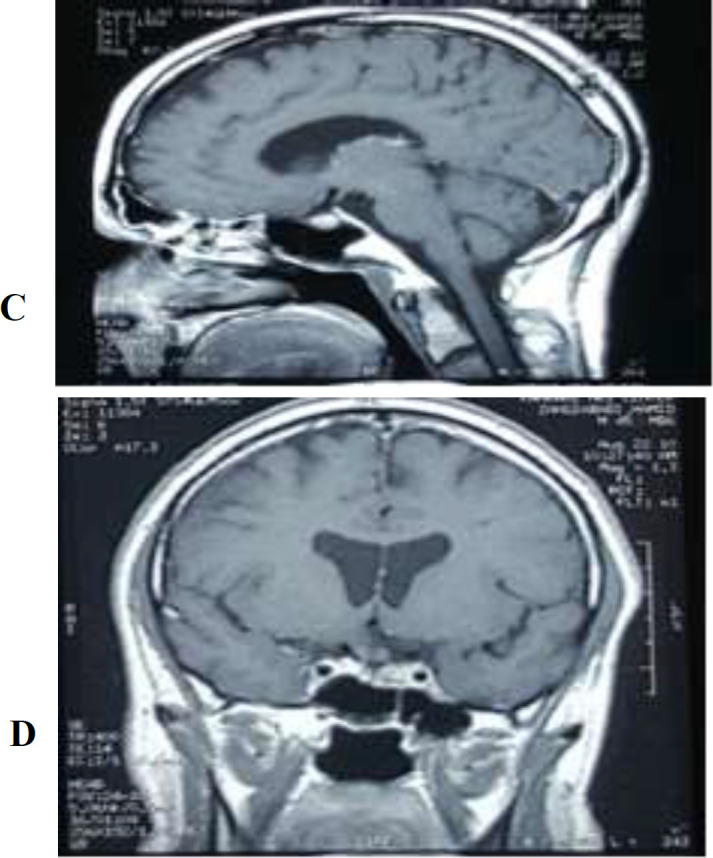
Postoperative T1 imaging (MRI) (sagittal C, coronal D, view) demonstrating a tumor residual in the left cavernous sinus

A 51-year-old man referred to the neurosurgery and endocrinology clinic (Loghman Hakim Hospital, Shahid Beheshti University of Medical Sciences, Tehran, Iran) who was a known case of pituitary macroadenoma, treated 6 years ago. Patient’s problems began 6 years ago while he suddenly developed following double vision, blurred vision and left eyelid ptosis. His primary lab tests showed a normal hormonal profile. Magnetic resonance imaging (MRI) revealed an invasive pituitary macroadenoma extended to suprasellar and compressing the optic chiasm, infrasellarly into the sphenoid sinus, and laterally invading into the left cavernous sinus (figure 1). He underwent trans-sphenoidal surgery (TSS) with the diagnosis of non- function pituitary macroadenoma. Patient's problem (diplopia and blurred vision) improved after the surgery. Postoperative brain MRI was ordered that reported “ small residue tumor in the left side of pituitary fossa. There is some erosion of posterior clinoid by it. The pathologic investigation revealed “the neoplasm composed of rather monomorphic cells, arranged in nests in a hemorrhagic stroma. The neoplastic cells have round to oval nuclei. Some prominent nucleoli and various amounts of acidophilic cytoplasm and scant mitotic figures are also seen”. The pathologic study made the diagnosis of silent “pituitary adenoma” according to IHC study reported up to 3-4% of tumor cells were positively stained for a proliferation marker, Ki-67 antigen and positive GH, FSH, ACTH and prolactin markers. The LH and TSH markers were negative. 2 months after the TSS surgery, a gamma knife radio-surgery was done for the patient due to tumor residues in left cavernous sinus (figure 2).

**Figure 3:**
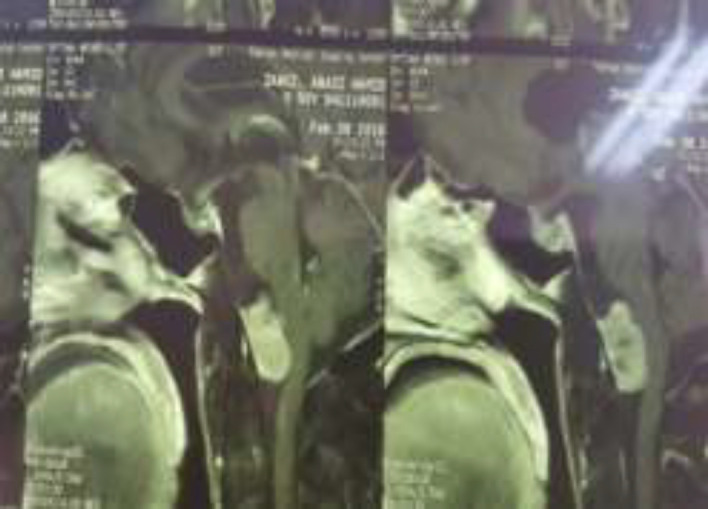
Preoperative T1 imaging (MRI) shows enhancing mass in the craniocervical junction (Ant cistern of brain stem) with compressive effect on ant medulla

He was diagnosed as being treated and asked to make follow-up visits which he neglected then. After 6 years, he made a visit to neurosurgery and endocrinology clinic with the chief complaint of weight gain, facial and extremities swelling, paresthesia, weakness, motion and speaking impairments so HTN which all appeared through the last 1 year. Routine blood tests with the suspicion of Cushing syndrome were done. Hormonal laboratory tests showed urine free cortisol 197.8 and 367. 30 ug/24hrs (36 -137), cortisol 8 am after 1 mg overnight dexamethasone 375 ng/mL (50-250) and ACTH 59 pg/mL. During MRI study, they found out about a mass in the brainstem with compressive effect on spinal region (figure 3), pituitary imagine does not differ from the last MRI (figure 2).

**Figure 4:**
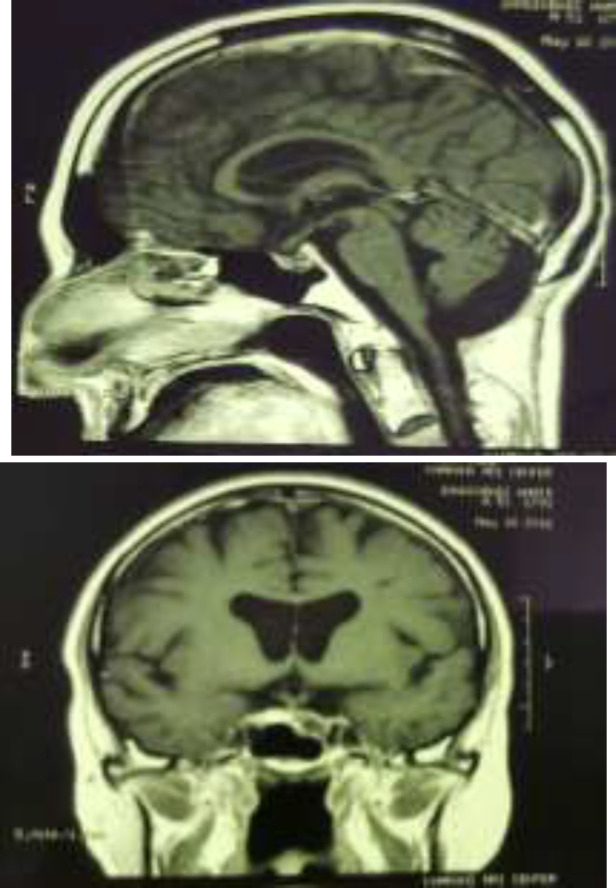
Postoperative T1 coronal and sagittal MRI after total mass resection in the second surgery

He underwent a neurosurgery for spinal mass resection, which was successful and the total mass was resected (figure 4). 

After surgery, symptoms of patient diminished and the general condition got better. The spinal specimen was sent for pathology which showed “malignant high-grade neoplasm with epithelioid features suggestive of carcinoma (primary from pituitary or metastatic) or other metastatic neoplasms (melanoma, sarcoma with epithelioid features). IHC markers were requested which showed positive values for CKAE1/AE3, CK7, CK20 and focally positive S-100 and positive ACTH marker was reported as metastatic carcinoma according to IHC findings.

An abdominopelvic CT-scan was ordered for the patient considering IHC results which were normal. For further assurance, a Tc-Octreotide scan was ordered which reported “no remarkable Tc-octreotide avid lesion was detected through the body (no somatostatin receptor positive lesion). Chemotherapy was begun for him 1 month after the surgery. His last laboratory exams after the chemotherapic treatment revealed IGF-1(insulin growth factor-1) 194 ng/mL (97-292), UFC 118 mg/24 hrs (50-190), cortisol 8am 6.37 mcg/dL(6.2-19.4), normal thyroid function tests, LH 3.74 mlu/mL, FSH 4.47 mlu/ml (1.5-12.4), ACTH 32.34 pg/ml (7.2-63.3), prolactin 25.90 ng/mL (1-21), testosterone 1.34 ng/mL (2.8-8.8). 

## Discussion

Pituitary carcinomas are rare, show cerebrospinal or systemic metastasis (6). Early detection of pituitary adenoma at risk for progression to the pituitary carcinoma is difficult and cannot be diagnosed by histopathology or pituitary imaging (6). In this patient which showed an up to 3-4% of tumor cells were positively stained for ki67 antigen, due to 2004 WHO classification, our tumor was an atypical adenoma, but current WHO criteria have abandoned grading tumors atypical due to its limited prognostic value (7). Aggressive pituitary tumor can be considered in this patient with a radiologically invasive tumor and relevant tumor growth despite surgery and radiotherapy (8). The role of previous radiotherapy in malignant transformation has been reported (9, 10). In our case report, primary invasive silent pituitary adenoma after gamma knife treatment showed cerebrospinal metastasis suspecting that radiotherapy may induce malignant transformation.

In this case-report, patient was treated primarily with TSS surgery after he had undergone gamma-knife surgery to remove all the remaining of the tumor. But unfortunately, due to the aggressive behavior of the tumor ,metastasis happened to the spinal region. As we were completely oriented that we were dealing with a primary pituitary macroadenoma, that was invasive silent corticotroph adenoma based on radiological and pathological conditions without clinical presentation of hypercortisolism at first, and emphatically after spinal mass resection ,the features of hypercortisolism improved and he got better. 

In the analysis performed by Yoo et al., CNS metastasis in 58.3% and systemic metastasis in 31.9% were reported in the pituitary carcinoma (11). Similar to our case report, David J et al, reported a 1.17 year old female, first presented with an invasive and clinically non functioning pituitary adenoma and then presented with Cushing disease and bone metastasis (12). Corticotroph pituitary carcinomas are the second most commonly encountered carcinomas. Most commonly (64% of ACTH carcinomas) corticotroph tumors secrete ACTH, and patients exhibit typical features of hypercortisolism( 13-15 ) that was compatible with our case. In the case presented herein, the interval between primary tumor to metastasis was 5-6 years after the initial diagnosis. The interval for progression to carcinoma has been reported to range from a few months to 32 years (13,15,16 ). It is usually really hard to diagnose the carcinoma before its invasion to the surrounding areas. Invasion of surrounding structures by pituitary tumors complicates complete resection and is an important cause for postoperative recurrence (17). Their prognosis is poor and about 80% of these patients die within 8 years (13). 

In conclusion we presented an uncommon case of corticotroph secreting pituitary carcinoma metastasized to the spine with the clinical presentation of hypercortisolism, the time of metastasis in the patient with a history of earlier resection invasive pituitary adenoma without clinical presentation of hypercortisolism (asymptomatic Cushing disease) simultaneously. Clinical awareness of the rare possibility for aggressive adenomas progresses, metastasis is essential to appropriately monitor patients for the possible early detection and treatment of pituitary carcinoma. We offer other researchers to do more research on this area of treatment and thankful for any share.

## References

[1] Meij BP, Lopes MB, Ellegala DB, Alden TD, Laws ER (2002). The long-term significance of microscopic dural invasion in 354 patients with pituitary adenomas treated with transsphenoidal surgery. J Neurosurg.

[2] Hansen TM, Batra S, Lim M (2014). Invasive adenoma and pituitary carcinoma: a SEER database analysis. Neurosurg Rev.

[3] Scheithauer BW, Kurtkaya-Yapicier O, Kovacs KT, Young WF Jr, Lloyd RV (2005). Pituitary carcinoma: a clinicopathological review. Neurosurgery.

[4] Kaltsas GA, Nomikos P, Kontogeorgos G, Buchfelder M, Grossman AB (2005). Clinical review: diagnosis and management of pituitary carcinomas. J Clin Endocrinol Metab.

[5] Ragel BT, Couldwell WT (2004). Pituitary carcinoma: a review of the literature. Neurosurg Focus.

[6] Daita G, Yonemasu Y (1996). Dural invasion and proliferative potential of pituitary adenomas. Neurol Med Chir (Tokyo).

[7] Lopes MBS (2017). The 2017 World Health Organization classification of tumors of the pituitary gland: a summary. Acta Neuropathologica.

[8] Colao A, Savastano S (2011). Medical treatment of prolactinomas. Nature Rev Endocrinol.

[9] Casson IF, Walker BA, Hipkin LJ (1986). An intrasellar pituitary tumour producing metastases in liver, bone and lymph glands and demonstration of ACTH in the metastatic deposits. Acta Endocrinol (Copenh).

[10] Tanaka T, Kato N, Aoki K (2013). Long-term follow-up of growth hormone-producing pituitary carcinoma with multiple spinal metastases following multiple surgeries: case report. Neurol Medi Chir.

[11] Yoo F, Kuan EC, Heaney AP, Bergsneider M, Wang MB (2018). Corticotrophic pituitary carcinoma with cervical metastases: case series and literature review. Pituitary.

[12] Holthouse DJ, Robbins PD, Kahler R, Knuckey N, Pullan P (2001). Corticotroph pituitary carcinoma: case report and literature review. Endoc Pathol.

[13] Pernicone PJ, Scheithauer BW, Sebo TJ (1997). Pituitary carcinoma: a clinicopathologic study of 15 cases. Cancer.

[14] Sheldon WH, Golden A, Bondy PK (1954). Cushing's syndrome produced by a pituitary basophil carcinoma with hepatic metastases. Am J Med.

[15] Scheithauer BW, Fereidooni F, Horvath E (2001). Pituitary carcinoma: an ultrastructural study of eleven cases. Ultrastruct Pathol.

[16] Takeuchi K, Hagiwara Y, Kanaya K (2014). Drop metastasis of adrenocorticotropic hormone-producing pituitary carcinoma to the cauda equina. Asian Spine J.

[17] Oruçkaptan HH, Senmevsim O, Ozcan OE, Ozgen T (2000). Pituitary adenomas: results of 684 surgically treated patients and review of the literature. Surg Neurol.

